# 1420. Optimizing the detection of environmental fungal contamination by comparing sample collection and detection methods

**DOI:** 10.1093/ofid/ofad500.1257

**Published:** 2023-11-27

**Authors:** Bobby G Warren, Amanda M Graves, Aaron Barrett, Alicia Nelson, Matthew Stiegel, Becky A Smith, Ilan Schwartz, Deverick J Anderson

**Affiliations:** Duke University School of Medicine, Hillsborough, North Carolina; Duke University School of Medicine Duke Center for Antimicrobial Stewardship and Infection Prevention, Durham, North Carolina; Duke Health, Cary, North Carolina; Duke University School of Medicine, Hillsborough, North Carolina; Duke University, Durham, North Carolina; Duke University, Durham, North Carolina; Duke University, Durham, North Carolina; Duke Center for Antimicrobial Stewardship and Infection Prevention, Durham, North Carolina

## Abstract

**Background:**

The use of environmental sampling to surveil the healthcare environment for invasive fungal species to prevent or respond to outbreaks is limited due to the absence of established threshold values and standard practices. This study aims to fill this gap by comparing sampling and detection methods in a controlled experimental setting.

**Methods:**

We compared the sampling efficacy of commonly used healthcare sampling techniques and the detection efficacy of culture-based and qPCR quantification on common healthcare surface materials. The primary outcome was recovery of organisms, defined as total recovered CFU compared to known inoculum CFU.

Mock 10x10cm study surfaces (aluminum, formica, linen, and HEPA material) were inoculated with ∼10^4^ CFU of *Aspergillus fumigatus* or *Candida parapsilosis* and allowed to air dry. Foam sponges and flocked swabs pre-moistened with neutralizing buffer were used to sample the complete surface area of surfaces. RODAC plates filled with species-specific media were pressed to the middle of the study surface and removed. Sponges were processed using the stomacher technique and swabs were vortexed in PBS. Eluents from sponges and swabs were used for both culture-based quantification and qPCR using FungiQuant primers and probe for the fungal 18S rRNA gene.

**Results:**

20 experiments were completed for each combination of species (n=2), study surface (n=4), sampling method (n=3) and quantification method (n=2) resulting in 960 total samples. Overall, median percent recovery for culture-based and qPCR-based detection methods were 6.4% (IQR: 2.8-12.9) and 26.7% (10.5-48.1), respectively, (p< 0.01). Median percent recovery for sponges and swabs were 17.9% (11.4-30.0) and 3.8% (1.9-6.7) via culture (p< 0.01) and 36.2% (25.7-78.4) and 10.5% (7.7-36.0) via qPCR (p< 0.01). RODAC median percent recovery via culture was 3.4% (1.0-7.1).

Fungal Recovery Characteristics by Fungal Species and Sample and Detection Methodology

**Figure:**
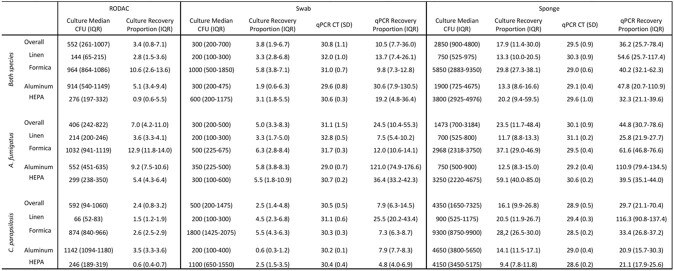

**Conclusion:**

qPCR-based detection had the highest median percent recovery compared to culture-based detection overall, regardless of sample method or study surface. Sponge samples had the highest median percent recovery for both culture- and qPCR-based detection methods, overall. Future studies are needed to assess our study’s identified optimized sample collection and detection techniques in a real-world healthcare environment.

**Disclosures:**

**All Authors**: No reported disclosures

